# Psychopathology, cortisol and testosterone responses to traumatic images: differences between urban and suburban citizens in a middle-income country

**DOI:** 10.3389/fpsyg.2023.1187248

**Published:** 2023-07-07

**Authors:** Lilian Mayagoitia-Novales, Ana Lilia Cerda-Molina, Sheila Adriana Mendoza-Mojica, Javier I. Borráz-León, M. Alejandra Hernández-Melesio, Gabriela Josefina Saldívar-Hernández

**Affiliations:** ^1^Departamento de Etología, Dirección de Investigaciones en Neurociencias, Instituto Nacional de Psiquiatría Ramón de la Fuente Muñiz, Ciudad de Mexico, Mexico; ^2^Unidad Académica Profesional Tejupilco, Universidad Autónoma del Estado de México, Estado de Mexico, Mexico; ^3^Institute for Mind and Biology, The University of Chicago, Chicago, IL, United States; ^4^Dirección de Investigaciones Epidemiológicas y Sociales, Instituto Nacional de Psiquiatría Ramón de la Fuente Muñiz, Ciudad de Mexico, Mexico

**Keywords:** cortisol, testosterone, psychopathology, urbanization, mental health, HPA axis

## Abstract

**Background:**

Living in urban places has been associated with a higher risk of psychopathology as well as with altered hypothalamus–pituitary–adrenal (HPA) axis and consequently altered cortisol response, but studies have concentrated mainly in high-income countries population. The role of other hormones such as testosterone, implicated in stress response and with human social behaviors, have not yet been investigated. The aim of this study was to compare symptoms of psychopathology as well as cortisol and testosterone in response to traumatic images between urban and suburban people in a middle-income country.

**Methods:**

A sample of 67 women and 55 men (*N* = 122, 18–45 years) from urban and suburban places of Mexico participated in the study. We quantified salivary cortisol and testosterone in response to images with traumatic and violent content (basal, 15, 30, and 45 min after images). Participants answered a general information questionnaire and the Symptom Checklist-90-R to assess their psychopathological traits. We performed Generalized Estimating Equation Models to analyze hormonal levels and MANOVAs to compare differences in participants’ psychopathology symptoms. Area under the curve respect to ground (AUC_G_) of hormonal levels and sex differences were also compared.

**Results:**

Suburban citizens showed no cortisol response, whereas urban people showed a cortisol peak 15 min after the image’s exposure; however, suburban people had higher AUC_G_ and basal levels compared to urban ones. Contrastingly, testosterone levels declined in all participants excepting the urban women, who showed no testosterone response. Although similar testosterone profile, AUC_G_ levels were higher in urban than suburban men. Participants living in suburban areas had higher scores of somatizations, obsessive–compulsive, and interpersonal sensitivity, as well as more sleep disorders than participants living in urban areas.

**Conclusion:**

This study offers novel evidence about differences in cortisol and testosterone responses to a social stressor and in mental health indicators between a population of urban and suburban citizens, highlighting the impact of urbanization process on physiological and psychological outcomes in a middle-income country.

## 1. Introduction

The impact of stress-related mental health diseases in people living in urbanized cities has been widely recognized (Dye, 2008; Peen et al., 2010; Alcock et al., 2014; Maxwell et al., 2021). The rapid urbanization process has persuaded many people living in rural communities to move into cities, since urbanization entails several advantages over rural areas, for example, better employment opportunities, higher incomes, better nutrition and education, primary health services, and more entertainment options (Peen et al., 2010). However, one of the main outcomes of this process is exposure to many social stressors, such as crowding, traffic, longer working hours, pollution, noise, and less exposure to green spaces, which negatively impact individuals’ mental and physical health (Dye, 2008; Alcock et al., 2014; Engemann et al., 2020). Previous literature has consistently reported that living in urbanized environments may be a risk factor for the development of some stress-related psychiatric disorders (Romans et al., 2011; Maxwell et al., 2021). For instance, some studies have documented a higher prevalence of major depressive disorder, schizophrenia, bipolar disorder, and non-affective psychosis in people living in urbanized cities (Romans et al., 2011; Vassos et al., 2012, 2016). Besides, it has been reported an increased brain amygdala activity associated with social stress processing in city dwellers compared to town or rural dwellers (Lederbogen et al., 2011). However, differences in mental health of people living in urban and rural areas among high-, middle-, and low-income countries have not been consistently described. Most rural citizens in low- and middle-income countries are still engaged in agricultural activities, without the technological developments to facilitate production, coupled with low levels of access to comprehensive health and education coverage; thus, rural families often live in constant financial uncertainty and poverty; these low socioeconomic conditions have been commonly linked to social stressors such as violence and crime scenarios (Lund et al., 2010).

On the other hand, it has been widely documented that experiencing traumatic and stressful life events can result in altered reactivity of the neuroendocrine stress response system, the hypothalamic–pituitary–adrenal (HPA) axis (McEwen and Seeman, 1999; Herman et al., 2016; Agorastos et al., 2019). Together with limbic structures such as the amygdala and hippocampus, an optimal functioning of the HPA axis is required for the expression of appropriate stress responses. The first pathway of stress response is driven by the activation of corticotrophin releasing hormone (CRH) from the hypothalamic paraventricular nucleus (PVN), which results in the release of adrenocorticotropic hormone (ACTH) from the hypophysis, and finally in glucocorticoids (GCs) secretion (corticosteroids or cortisol in humans) from the adrenal glands (Anacker et al., 2011; Herman et al., 2016). All this process temporarily interrupts the metabolic homeostasis of the body; however, GCs also act in peripheral organs such as the liver to mobilize energy reserves, i.e., glucose, to maintain the body homeostasis and adaptation on to environmental demands (Sapolsky et al., 2000). Once the stressor is over, GCs activate a negative feedback mechanism by inhibiting further HPA axis activity; alterations in this regulation system might provoke abnormally high or low GCs secretion (Joseph and Golden, 2017). Individuals might habituate to chronic psychosocial stressors by decreasing the HPA axis activation to promote adaptation to challenges and to protect the body from the negative effects of prolonged GCs secretion (Wüst et al., 2005). Another steroid hormone secreted during challenging situations is testosterone (Chichinadze and Chichinadze, 2008; Borráz-León et al., 2019), a reproductive hormone synthesized in higher concentrations in the Leydig cells of males and lower concentrations in the ovaries of females, as well as in the adrenal glands in both sexes (Baird et al., 1969). The nature of testosterone response varies according to personality traits and contexts, e.g., whether the situation represents a social threat, a stressor, or an anticipation of a challenge (Chichinadze and Chichinadze, 2008; Bedgood et al., 2014). For instance, Bedgood et al. (2014) reported that young adult men with lower basal cortisol levels had higher increases in testosterone levels after a social evaluative stressor, which might prepare the body for competitive contexts. Although there is evidence indicating a heightened vulnerability to mental illness as well as a higher stress response in people raised in cities than in small towns or rural areas, this information mainly comes from high-income countries [e.g., Germany (Lederbogen et al., 2011; Steinheuser et al., 2014), Netherlands (Peen et al., 2007, 2010), US (Pun et al., 2019), Denmark (Engemann et al., 2020), UK (Maxwell et al., 2021)], restricting its generalizability to other populations with different socioeconomic status. To the best of our knowledge, studies on alterations in the HPA axis reactivity and their association with mental health indicators in low- and middle-income countries are very scarce, where non-urban citizens may be affected by stressors such as poverty and violence.

In this regard, Mexico is a middle-income country (WHO, 2003) characterized by being a heterogeneous country, both in population and territory, as it has faced an economic growing progress with regional disparities and inequalities. In contrast to the rural and suburban (i.e., in process of urbanization) regions of the country, the big urban cities, such as Mexico City, the capital of the country (with around 9 million inhabitants; Instituto Nacional de Estadística y Geografía, 2020), has greater access to resources and services, e.g., health care system, education, more entertainment options, and higher paying jobs (COESPO, 2019). However, urban citizens might live in scenarios where insecurity and violence are common without advertising the magnitude of the problem because of the population density. For instance, urban areas are so crowded and complex that their inhabitants are not always aware of the different forms of violence, direct (being a victim) or indirect (e.g., witnessing, watching, or hearing the news), occurring in a single day (e.g., assaults, homicides, or femicides). Contrastingly, in rural and suburban areas, the levels of violence (mostly direct) vary from those in urban areas, but the lower population density increases the perception of insecurity (Robles et al., 2019). Witnessing a crime can be distressing, particularly to those people who has also been a victim of a crime (mild or severe) (Kennedy and Ceballo, 2014). In fact, previous literature has reported that mental health consequences for victims of direct and indirect violence are mostly the same (Lorenc et al., 2012).

Unfortunately, the insecurity issues exceed the authority’s capability to adequately manage them (Índice de Paz México, 2020). Then, the insecurity perception and the uneven distribution of progress that face rural/suburban citizens in marginalized areas, contribute to a reduced self-perception of well-being. In contrast to the Capital of the Country, other cities such as the State of Mexico (a geographical region adjacent to the capital) is characterized for being one of the most violent areas in the country with the highest national crime incidence including kidnappings, extortion, drug trafficking, and the highest rate of feminicides in the Country (Instituto Nacional de Estadística y Geografía, 2020; Índice de Paz México, 2020). Despite being one of the most populated regions in the Country, the State of Mexico has many contrasting small suburban communities (whose main economic activities are manufacturing, mining, and agricultural industry), some of them with less than 50,000 inhabitants and others with less than 5,000 inhabitants. Compared to Mexico City, people living in those small communities have little access to basic services such as public transportation or specialized health care services, which sometimes are far away from their places of residence (Secretariado Ejecutivo del Sistema Nacional de Seguridad Pública, 2016; Mendoza-Mojica et al., 2017).

Therefore, the goal of the present study was to compare cortisol and testosterone secretion in response to images with violent and traumatic content as well as symptoms of psychopathology and psychological distress between people living in an urban big city and suburban communities. Considering that prolonged exposure to violent scenarios could create an emotional desensitization process (Fanti et al., 2009), we hypothesized that people living in the suburban communities will have a reduced cortisol response (i.e., blunted, or flattened reactivity) reflecting a process of habituation and an increased number of distress symptoms signaling poorer mental health compared to people living in the urban city (with better security perception). Besides, since individual differences in stress reactivity might depend on how people appraise adversity -as challenges or social threats- we expected an inverse relationship between testosterone and cortisol secretion.

## 2. Materials and methods

### 2.1. Participants

Participants between the ages of 18 and 50 years were invited to participate in the study. The sample included a non-clinical population of 122 adults, 67 women and 55 men (mean age = 29.81 [SD = 6.99], range 18–45). Exclusion criteria were: substance abuse, serious physical illness, current respiratory illness, not being diagnosed with any psychiatric illness currently or within the last 12 months, those conditions affecting cortisol levels such as pregnancy, using birth control pills, and/or current treatment with psychiatric or anti-inflammatories drugs. Fifty-five volunteers were University workers and students living in Mexico City (an urban place), and 67 were workers and students from The College of Scientific and Technological Studies of the State of Mexico (living in suburban surrounding communities; Instituto Nacional de Estadística y Geografía, 2020; see Table 1 for demographic data). These geographic areas were chosen due to their contrasting sociodemographic characteristics in terms of income opportunities, access to public transportation, specialized research and health care centers, and security systems. While Mexico City is a crowded place, with better sociodemographic advantages, the suburban communities of the State of Mexico are much less crowded, but have more limited access to those advantages, situation that drug trafficking networks mostly take as advantageous, making people feel insecure.

**Table 1:** Demographic characteristics of the population.

	Urban (*N* = 55)	Suburban (*N* = 67)	Test	Sig.
Age	24.73 (5.82) R (18–45)	33.99 (4.75) R (24–43)	*t* = − 9.47	< 0.001
BMI	24.24 (3.20) R (18.83–30.78)	27.43 (4.04) R (19.38–37.04)	*t* = − 4.86	< 0.001
Smoke	No = 42 Yes = 13	No = 56 Yes = 11	χ^2^ = 0.99	0.31
Relationship status	*S* = 21 *R* = 34	*S* = 17 *R* = 50	χ^2^ = 0.16	0.09
Education level			χ^2^ = 5.90	0.052
High school	*N* = 2	*N* = 5		
University	*N* = 45	*N* = 60		
Master	*N* = 8	*N* = 2		

Data express the mean, standard deviation of the mean (SD) and range (R) as well as the frequency.

BMI, body mass index; Relationship status: S, single; R, in a relationship.

### 2.2. Ethics statement

This study has the approval of the Research and Ethic Committees (Project Number NC17076.0). The procedure adheres to the declaration of Helsinki as well as the National Official Norms for Research with Human Beings (NOM-012-SSA3-2012); all the volunteers gave their informed consent, had the right to finish the procedure at any time, and no identifying information was collected. The manuscript does not contain clinical studies or patient data.

### 2.3. Study design

All volunteers who agreed to participate signed a consent form explaining the procedure and purpose of the study while ensuring the confidentiality of their information. Volunteers were carefully instructed to brush their teeth before coming to the test and not to smoke, eat or drink anything except water for at least 2 h before completing saliva sampling. All sessions took place between 11:00 and 14:00 h in a closed but ventilated room. Participants completed a general information questionnaire and the Symptom Checklist-90-R (SCL-90-R); after that, volunteers were asked to donate a first saliva sample (2–3 mL) via passive drool, into a new polypropylene tube (15 mL) which were labeled as basal samples. After the basal sample was collected, participants were visually exposed to 12 standardized images (shown in a flat screen) with violent and traumatic content taken from the International Affective Picture System (Lang et al., 1997), which have been previously used in Mexican population (Reyes-Mota et al., 2021). Images used in this study and code numbers according to the IAPS were: 3001, 3,015, 3,016, 3,030, 3,053, 3,060, 3,080, 3,103, 9,405, 9,410, 9,921, 9,419. The images were shown in a Power Point presentation automatized for 5 s each one with a resting period of 10 s between each of them. We collected saliva samples after 15, 30, and 45 min of the onset of the visual stimulation. All the procedure took around 60 min. Saliva samples were labeled with a code to ensure the confidentiality of the volunteers and immediately frozen and stored at −20°C until assayed. This cross-sectional study was conducted between October 2019 and February 2020, prior to the COVID-19 pandemic which discards the effects of the pandemic on our results.

### 2.4. Measures

#### 2.4.1. Psychopathological symptoms

To assess mental health status, we applied The Symptom Checklist-90-Revised (SCL-90-R) a self-report scale that measures the degree of psychological distress and symptoms of psychopathology of both clinical and non-clinical populations (Derogatis and Fitzpatrick, 2004). The scale was validated for Mexican population by Cruz-Fuentes et al. (2005), with Cronbach’s alphas ranging from >0.7 to 0.85. SCL-90-R consisted of 90 items grouped into nine dimensions: somatization, obsessive–compulsive, interpersonal sensitivity, depression, anxiety, hostility, phobic anxiety, paranoid ideation, and psychoticism. Responses were measured using a Likert scale: 0 = none, 1 = mild, 2 = moderate, 3 = severe, and 4 = very severe. We added the answers of every item to obtain the total score of each dimension. We also analyzed seven items that are not incorporated into the 9 dimensions but that might reflect additional distress symptoms: poor appetite, trouble falling asleep, awakening in the early morning, sleep that is restless or disturbed, thoughts of death or dying, overeating, and feelings of guilt. Cronbach’s alphas range for the present sample: 0.78–0.91.

#### 2.4.2. Processing of saliva samples and hormonal measurements

Samples were subjected to two subsequent freeze–thaw cycles to free them from mucopolysaccharides and proteins. After each thawing, samples were centrifuged at 1500 g during 30 min at 4°C; the supernatants were collected and frozen again (Reyes-Mota et al., 2021). We measured salivary cortisol and testosterone in duplicates using commercially available kits for ELISA (ENZO life sciences) and by following the manufacturer’s instructions. For cortisol, inter- and intra-assay coefficients were 9.8 and 7.1% respectively, and for testosterone were 9.9 and 8.3%. Hormonal concentrations were reported in pg./mL.

### 2.5. Statistical design

Demographic data was compared by using *t* tests for independent samples and χ^2^. To analyze cortisol and testosterone responses to the images, we used Generalized Estimating Equation Models (GEE) appropriate for data dependency, i.e., repeated samples design (Pekár and Brabec, 2018). We performed three GEEs, one for cortisol levels and the other two for testosterone (broken out by sex). Hormone levels were introduced as dependent variables and as independent variables we introduced the type of city (Urban vs. Suburban), time (basal sample, 15, 30, and 45 min post-images), and sex (men-women, only for cortisol); age (in years) was introduced as covariate. We used Bonferroni as post-hoc test. Hormone levels were log-transformed to normalize the data. Additionally, we transformed hormonal levels into an area under the curve values (AUC_G_) by using the formula according to Fekedulegn et al. (2007). To compare AUC_G_ levels between cities we used a One Way ANOVA. Two Multivariate Analyses of Variance (MANOVA) were conducted to test differences in the SCL-90-R between cities, in the first one, we introduced the total scores of the 9 dimensions and in the second one the Likert scores of the seven additionally items as dependent variables; type of city and sex were introduced as independent variables. Spearman correlations were performed between basal levels of cortisol and psychopathology symptoms. Data were analyzed using SPSS for Windows version 21.0. The significance level was set at *p* ≤ 0.05 (bilateral).

## 3. Results

### 3.1. Demographics

Volunteers from the urban area were younger and had less BMI than the suburban volunteers (Table 1). We did not find statistically significant differences between places for the number of smokers, relationship status (i.e., in a relationship or single) or educational level, although a tendency for more participants with a master’s degree in the urban city was observed.

### 3.2. Cortisol response

**Figure 1:**
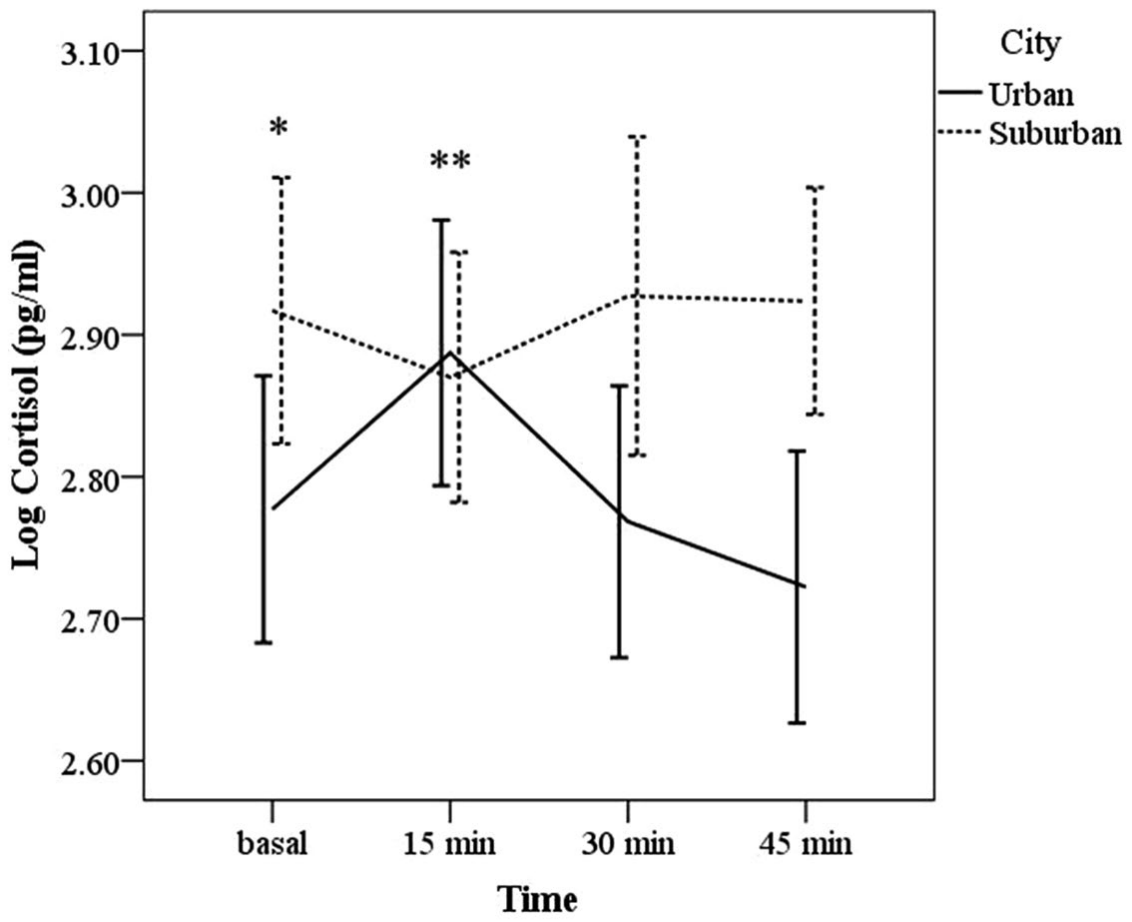
Mean (± CI 95%) of salivary cortisol levels (log transformed) before (basal), 15, 30, and 45 min after the exposure of images with violent content in people living in urban and suburban cities. Urban city ** *p* < 0.01, 15 min vs. 45 min; * *p* < 0.05, Suburban vs. Urban city.

The analyses showed that the interaction time × city was significant (χ^2^ Wald = 14.48, d.f. = 3, 122; *p* = 0.002); no effects were found for age or sex (χ^2^ Wald = 1.83, d.f. = 1, 122, *p* = 0.17; χ^2^ Wald = 0.008, d.f. = 1, 122, *p* = 0.93, respectively). Figure 1 shows that people from suburban cities had a flattened cortisol profile without significant changes over time (*p* ≥ 1.0 for all times). Contrastingly, a rise in cortisol levels was evident 15 min after the images in the urban citizens; however, this rise was not statistically significant compared to basal or 30 min levels (*p* = 0.36 and *p* = 0.37, respectively). Cortisol levels at 15 min after the images were statistically different from the levels at 45 min (*p* = 0.01). Basal cortisol levels were higher in suburban citizens compared to urban ones (*p* = 0.03). Although flattened response, AUC_G_ of people living in suburban cities was higher than the urban city (*F* = 4.38; *p* = 0.03; *M* = 4.60 [SD = 0.29] vs. *M* = 4.49 [SD = 0.28]).

### 3.3. Testosterone response

For women, the analyses showed that the interaction time × city was significant (χ^2^ Wald = 11.02, d.f. = 3, 67; *p* = 0.01) indicating that testosterone secretion was different between cities. We also found a tendency for age to be significant (χ^2^ Wald = 3.66, d.f. = 1, 67, *p* = 0.056).

**Figure 2:**
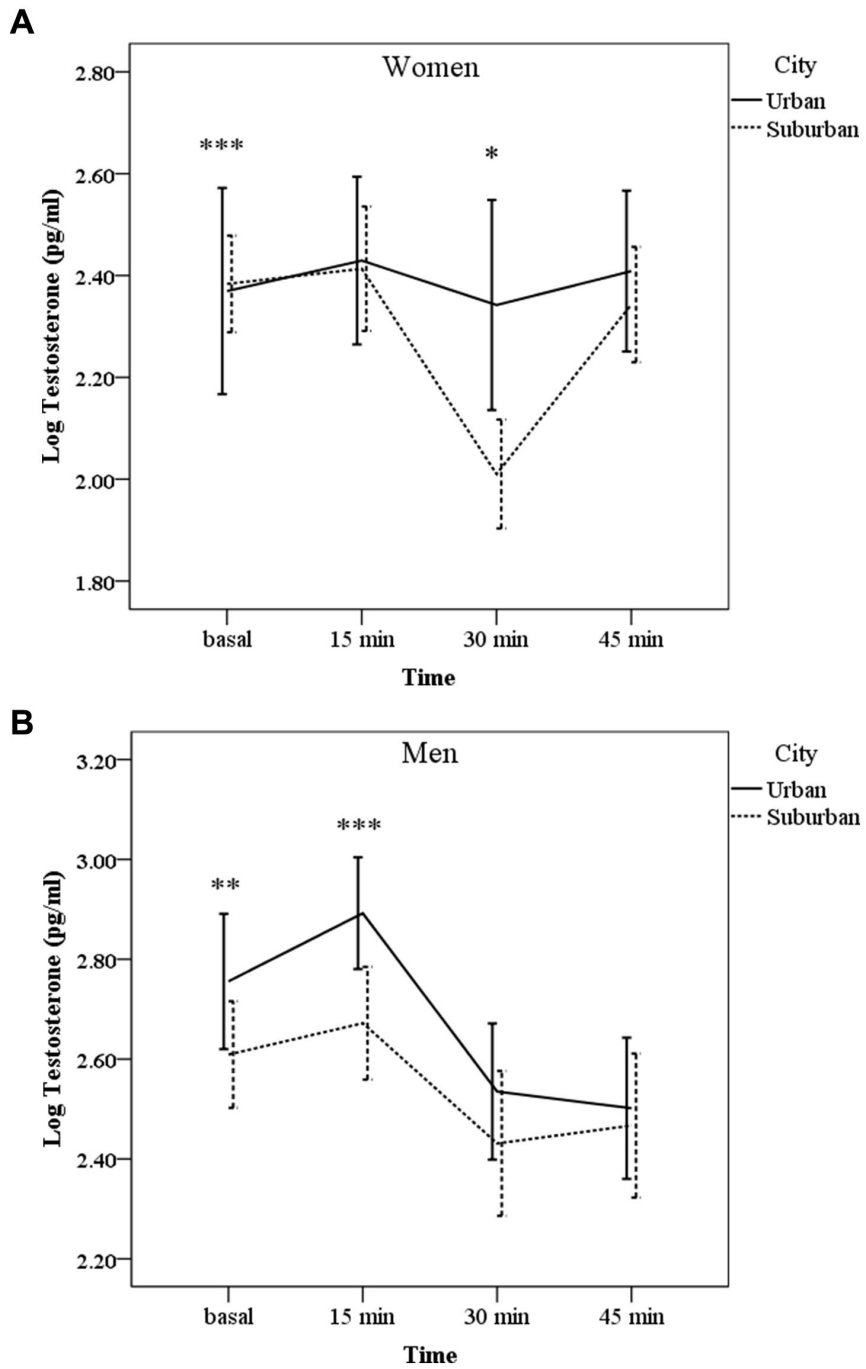
Mean (± CI 95%) of salivary testosterone levels (log transformed) before (basal), 15, 30, and 45 min after the exposure of images with violent content in people living in urban or suburban cities separated by sex. **(A)** Women: *p < 0.05 urban vs. suburban. ***p < 0.001 basal vs. 30 min in suburban samples. **(B)** Men: ***p < 0.001 vs. 30 and 45 min in both cities; **p < 0.01 vs. 30 and 45 min in both cities.

Figure 2A indicates no statistically significant testosterone changes over time in women from the urban city, whereas in suburban women testosterone decreased 30 min after the images compared to basal sample (*p* < 0.001) and compared to urban women at the same time (p = 0.01). AUC_G_ for testosterone did not differ between cities (*F* = 3.17, *p* = 0.07).

For men, neither the interaction time × city nor age were significant (χ^2^ Wald = 3.28, d.f. = 1, 55, *p* = 0.35; χ^2^ Wald = 0.73, d.f. = 1, 55, *p* = 0.39, respectively). City and time as main effect were significant (χ^2^ Wald = 7.34, d.f. = 1, 55, *p* = 0.007; χ^2^ Wald = 51.09, d.f. = 1, 55, *p* = 0.003, respectively). Figure 2B shows post-hoc comparisons for time; in both places, testosterone levels decreased 30 and 45 min after the image’s exposure compared to basal levels (*p* = 0.003 and *p* = 0.01, respectively) and to 15 min levels (*p* < 0.001). Although the profile of decrease was the same, AUC_G_ for testosterone in men’s urban volunteers was higher than in suburban ones [*F* = 12.69, *p* = 0.001; *M* = 4.30 (SD = 0.30) SD vs. *M* = 4.09 (SD = 0.28), respectively].

### 3.4. Psychopathological symptoms

**Figure 3:**
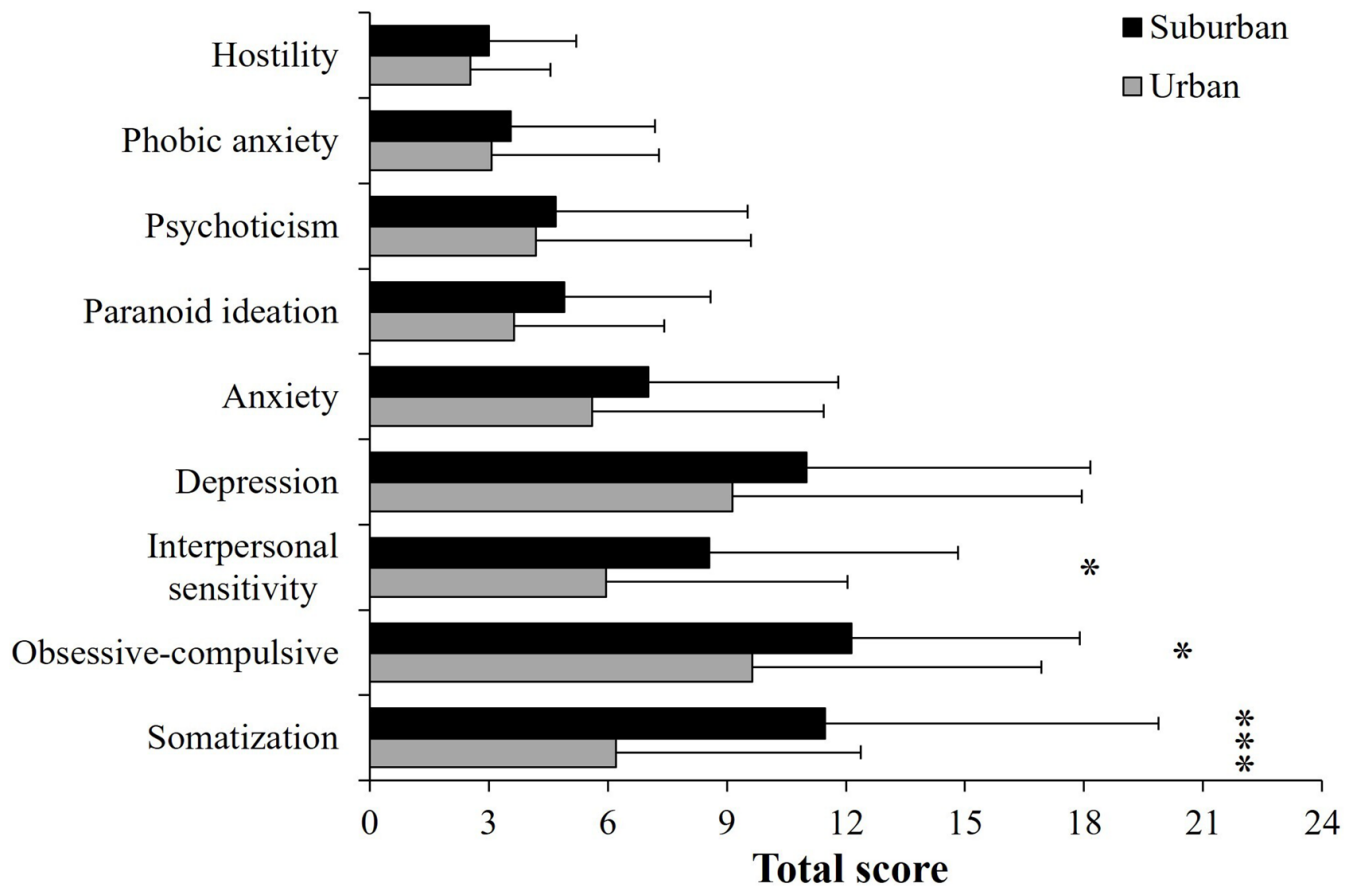
Mean (± SD) of the checklist symptoms (nine dimensions) of participants living in urban or suburban cities. ***p < 0.001; *p < 0.05.

**Figure 4:**
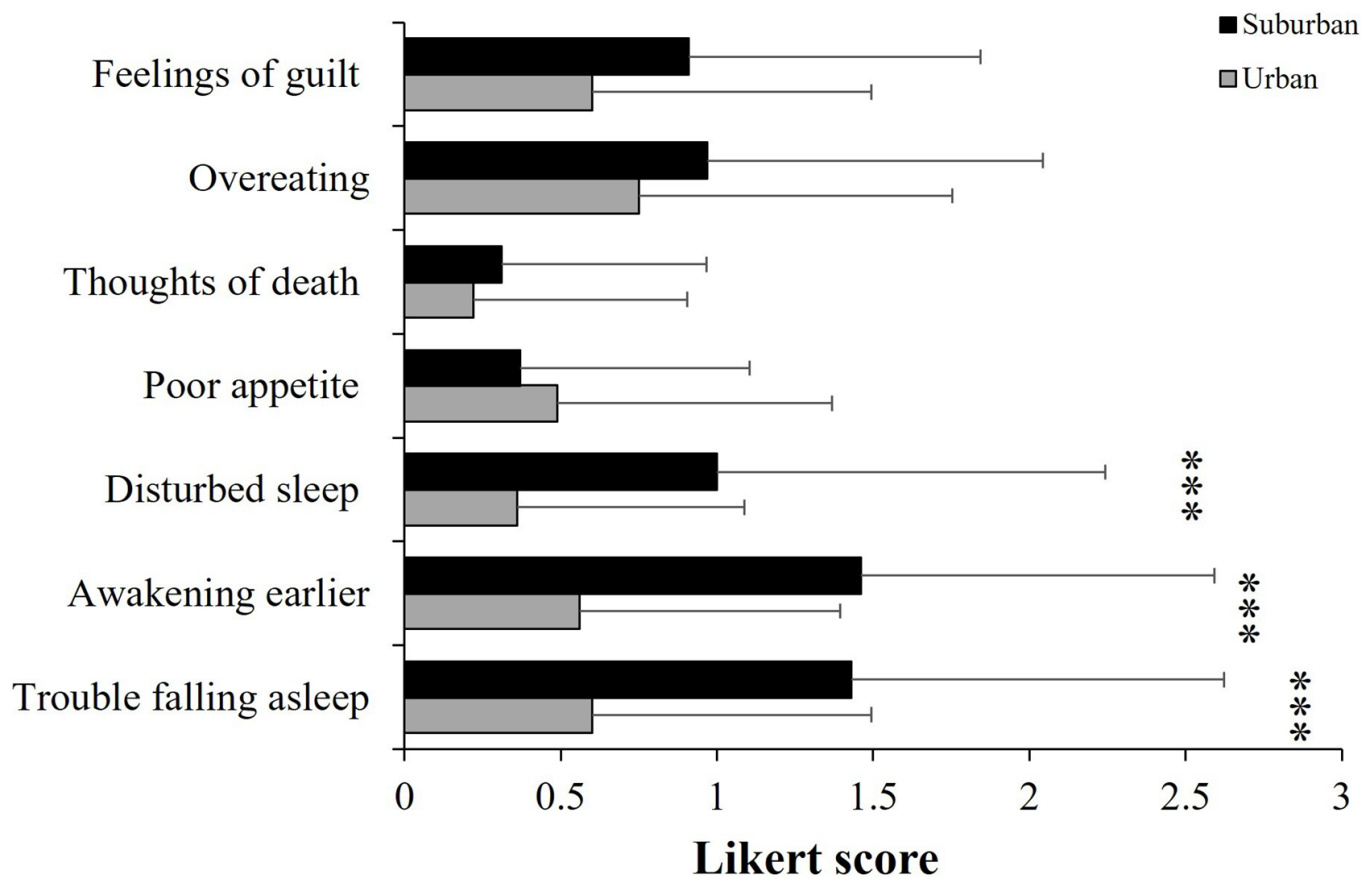
Mean (± SD) of the checklist symptoms (seven items) of participants living in urban or suburban cities. ***p < 0.001.

The MANOVA showed significant differences between cities for the 9 dimensions (*F* = 2.63, d.f. = 9,110, *p* < 0.001, *η*^2^ = 0.17), but not between sexes (*F* = 1.32, d.f. = 9, 110, *p* = 0.23, *η*^2^ = 0.09). Pair contrast analyses indicated that participants living in suburban cities had higher scores of psychological distress symptoms than urban participants (Figure 3); however, this difference was statistically significant only for Somatization (*p* < 0.001), Obsessive–Compulsive (*p* = 0.03), and Interpersonal Sensitivity (*p* = 0.02); a tendency for Paranoid Ideation (*p* = 0.06) was also found. The other dimensions were not statistically different between cities: Depression (*p* = 0.20), Anxiety (*p* = 0.14), Hostility (*p* = 0.43), Phobic Anxiety (*p* = 0.50), and Psychoticism (*p* = 0.58). Similarly, the MANOVA for the additional symptoms indicated significant differences between cities (*F* = 4.93, d.f. = 7,113, *p* < 0.001, *η*^2^ = 0.23), but not between sexes (*F* = 0.56, d.f. = 7,113, *p* = 0.78, *η*^2^ = 0.03). Suburban people also scored statistically higher in the following symptoms: trouble falling asleep, awakening in the early morning, and sleep that is restless or disturbed (*p* < 0.001 in all cases, Figure 4); the other symptoms were not different between cities: poor appetite (*p* = 0.48), thoughts of death or dying (*p* = 0.34), overeating (*p* = 0.26), and feelings of guilt (*p* = 0.07).

### 3.5. Psychopathological symptoms and basal cortisol levels

We found a significant negative association between basal cortisol levels and Obsessive–compulsive and Interpersonal symptoms in the urban participants (Table 2). On the opposite, we found significant positive correlations between depression, paranoid ideation, psychoticism, awakening in the early morning, and sleep that is restless or disturbed in the suburban population (Table 2).

**Table 2:** Spearman correlations between psychopathological symptoms (SCL-90) and basal cortisol levels in urban (*N* = 55) and suburban citizens (*N* = 67).

Symptom	Urban	Suburban	Symptom	Urban	Suburban
Somatization	−0.188	0.111	Trouble falling asleep	−0.001	0.003
Obsessive–compulsive	−0.280*	0.118	Awakening earlier	−0.020	0.291*
Interpersonal sensitivity	−0.281*	0.186	Disturbed sleep	0.031	0.274*
Depression	−0.150	0.247*	Poor appetite	0.041	0.205
Anxiety	−0.233	0.183	Thoughts of death	0.051	0.077
Hostility	−0.243	0.157	Overeating	0.064	0.075
Phobic anxiety	−0.227	0.073	Feelings of guilt	−0.190	0.130
Paranoid ideation	−0.162	0.247*			
Psychoticism	−0.198	0.306**			

*p < 0.05; **p < 0.01.

## 4. Discussion

We found that urban citizens exhibited a tendency to increased cortisol levels after the exposition of images with traumatic content followed by a rapid recovery, suggesting that the images represented a stressor, since cortisol is one of the HPA axis mediators released during a stress response (McEwen and Seeman, 1999). In contrast, although suburban citizens showed no significant response, they had higher basal and total levels of cortisol than urban people. In line with our hypothesis, this result could indicate that suburban residents might be habituated to live in more adverse environments and consequently, the images were not perceived as a real social threat. Violence normalization in everyday life, goes parallel with the false idea that violence only impacts the victim; however, what happens in a family, on the street, or at school usually impact whole society (Schmidt et al., 2018). Because the magnitude of the HPA response is mostly regulated by the emotions elicited by the situation, the lack of cortisol response we found in the suburban population may also be the manifestation of a fragmented social networks; when violence is normalized as part of the dynamics of a society, individuals are less critical of it, affecting their ability to trust others, weakening one of the most fundamental bases of community well-being and development (Zimmerman and Posick, 2016). Alternatively, having total elevated cortisol levels could indicate that the neuroendocrine HPA axis may be hyporesponsive, due to previous frequent activations that commonly alter the sensitivity of the negative feedback (Gunnar and Vazquez, 2001). Together with elevated basal cortisol levels, suburban citizens scored higher than urban people in several psychopathological symptoms primarily in somatization, interpersonal sensitivity, obsessive–compulsive, and those related with the quality of sleep. These findings support our hypothesis that living in suburban environments may be more stressful than living in urban cities, at least in our study population of a middle-income country. Our results also show the importance of studying vulnerable populations in which social problems such as marginalization or violence, cause serious mental health problems that affect quality of life, as well as personal and social relationships (Hollie and Coolhart, 2020; Gómez et al., 2021). Interpersonal interactions within societies are part of the psychological well-being of people; then, when interpersonal functioning changes to an interpersonal distress, many forms of psychopathology develop, for example, symptoms of depression, paranoid, schizoid, schizotypal or antisocial personality symptoms (Latkin and Curry, 2003; Elzinga et al., 2008).

Many suburban communities in Mexico are characterized for being in transition from rural to urban environments, where many families still depend on agricultural or informal jobs (with no social security). Besides, to get places such as High School, University, or Health Services, is not easy due to a deficient system of highways and public transportation, all of which may contribute to live in a context of cumulative stressful situations leading to flattened cortisol responses and a poor mental health, signaled by higher psychopathological symptoms. For instance, some authors have associated a reduced cortisol response with experiencing early life stressors, traumatic experiences, and with living in socio-economically disadvantaged neighborhoods (Brenner et al., 2013; Abell et al., 2016; Tsai et al., 2019). Contrastingly, elevated concentrations of cortisol have been associated with altered normal sleep pattern and with more frequent awakenings during sleeping hours (Ross and Jang, 2000; Myin-Germeys and van Os, 2007). Some other authors have described that adverse perceptions of the neighborhood affect physical and mental health of the residents by increasing fear and anxiety (Procyshyn et al., 2020). Most research on the effects of urbanization processes on people’s mental health, argues that challenging situations faced in cities, such as overcrowding, traffic, or noise, may be associated with the development of mental health disorders such as anxiety, major depressive disorder, and psychoticism (Romans et al., 2011; Vassos et al., 2012, 2016). Contrastingly, together with higher psychopathological and distress symptoms, our findings indicated a positive association between basal cortisol levels and depression symptoms, paranoid ideation, psychoticism, and altered sleep patterns in suburban citizens, supporting the notion of negative effects of increased cortisol concentration (Sapolsky et al., 2000; Wüst et al., 2005). Some stress-related mental disorders such as depression and psychotic disorders, are frequently associated with low assertiveness, contributing to the experience of stressful interpersonal circumstances that may reduce the coping abilities (Wirth and Schultheiss, 2007; Procyshyn et al., 2020).

Some other studies have argued that urban citizens are more challenged by social evaluative stressors than rural citizens (Lederbogen et al., 2011). However, the differences in cortisol and testosterone levels as well as in psychopathological symptoms in our populations might reflect that, whereas urban people might perceive their living environments as more socially challenging or competitive, suburban people might perceive its living places as more socially stressful. For instance, although in our study, people from the urban city are exposed to higher levels of noise, pollution, or artificial light at night, volunteers from the suburban communities expressed more sleeping problems than those living in the urban area. This might be due to the unpredictable insecurity environments, which is a key element for perceived stress, increasing vulnerability to mental health disorders among suburban residents. In line with this idea, some studies have reported an association between several poverty indicators in low- and middle-income countries, and common mental disorders such as anxiety and depression (Lund et al., 2010). However, it is difficult to state whether there are more stressors in suburban places or if the greater access to basic services or recreational spaces could buffer the stress perception in urban residents.

In the present study, we also explored testosterone response, and our findings indicated that urban men had higher testosterone levels than suburban men, although the response to the images were similarly decreased. According to Bedgood et al. (2014), testosterone responses depend on whether a specific situation is perceived as a social threat, a stressor, or represents an anticipation of a challenge. Since testosterone and other hormones such as oxytocin, modulates, at a brain level, empathy as well (Borráz-León et al., 2018), the observed decline of testosterone in response to the traumatic images, in both populations, might be the manifestation of an emotional fearful or empathic response. However, the higher androgen levels observed in urban citizens might contribute to maintain an increased state of vigilance and competitiveness, that implies living in a more crowded city, as it has been described by some other authors (Wingfield et al., 2001; Zilioli and Bird, 2017).

On the other hand, we found a different profile of testosterone in women; those living in suburban areas showed a pronounced testosterone decrease after the exposure to the images followed by a rapid recovery, in contrast with the no response of the urban women. It is possible that women in suburban areas, although did not show an HPA axis activation, a reduced testosterone response might indicate -similar to that described in men- a higher emotional sensitivity to the traumatic images than women living in urban areas. The decline of testosterone in the lack of a real threat or challenge, could be adaptive because it might offer protection against the physiological costs of showing chronic high testosterone levels such as a reduction of lifetime fitness and impairments in the immune system (Girard et al., 2017). Although the hypothalamic–pituitary-gonadal system is the main pathway responsible for androgen secretion in men and women, the adrenal gland is also a source of testosterone synthesis, suggesting an interaction with the activation of the HPA axis. Some authors have demonstrated rapid fluctuations in testosterone in response to social and challenging stimuli in humans; then besides gonadal production, other faster pathways, such as sympathetic activation of the adrenal gland also contributes to the secretion of the androgen (Wilson et al., 2017). In sum, this study offers novel evidence about differences in cortisol and testosterone responses to a social stressor between urban and suburban citizens in a middle-income country, where suburban people enjoy neither a rural healthy green environment nor the advantages of the urban cities, such as specialized medical services, safe housing, or effective transportation. These complex living environments are reflected in a lack of cortisol response to violent scenarios, and with a decreased testosterone concentration, a hormone with important effects on social interactions and emotions. Overall, the findings presented in this research highlight the importance of considering the characteristics of both, urban and suburban living regions as well as hormonal profiles in mental health studies using clinical and non-clinical subjects.

## 5. Conclusion

Our findings support the hypothesis of the existence of differences in cortisol and testosterone responses to a social stressor and in mental health indicators between a population of urban and suburban citizens in a middle-income country, highlighting the impact of urbanization process on physiological and psychological health. Given that the HPA axis functions as an important pathway by which social, environmental, and psychological factors influence biology and health, our findings contribute to the knowledge of one of the mechanisms that increase the risk for psychopathology in suburban populations. More studies are needed to explore sex differences in the response of testosterone to stressors and their impact on mental health. Besides, future research should explore and compared our findings with data collected from rural regions.

## 6. Limitations

There were some limitations in this study. Firstly, the small sample size of both, urban and suburban populations, which might not be representative of the country, limits the generalizability of the conclusions. It is important to point out that we did not continue the saliva samplings because of the COVID pandemics at the beginnings of March 2020. Despite the statistically significant differences in cortisol response between both populations, we had the limitation of not having a neutral or “no-stressing” images control, which might limit our interpretation of and altered sensitivity of the HPA axis. Besides, we did not contemplate variables such as socioeconomic status or the experience of early life stressors that could have explained some differences in psychopathological symptoms of the volunteers. Another variable that might have explained the observed differences between urban and suburban places is the violence and insecurity perception. Future research must take all these limitations into account to a better understanding about why in middle-income countries urban cities could be less stressful than suburban places.

## Data availability statement

The raw data supporting the conclusions of this article will be made available by the authors, without undue reservation.

## Ethics statement

The studies involving human participants were reviewed and approved by Comité de Ética en Investigación. The patients/participants provided their written informed consent to participate in this study.

## Author contributions

AC-M designed the proposal, contributed to the methodology, data analyses, and writing-original draft preparation. LM-N contributed to the proposal design, recruited the participants, measured the hormones, contributed to the methodology, review, and editing manuscript. SM-M recruited the participants, contributed to the application of the scales, and data curation. JB-L contributed to methodology, and writing-original draft preparation. MH-M contributed to data curation and literature systematic review. GS-H contributed to review and editing the manuscript. All authors contributed to the article and approved the submitted version.

## Funding

The research was funded by the Instituto Nacional de Psiquiatría Ramón de la Fuente Muñiz, Mexico City, Mexico.

## Conflict of interest

The authors declare that the research was conducted in the absence of any commercial or financial relationships that could be construed as a potential conflict of interest.

## Publisher’s note

All claims expressed in this article are solely those of the authors and do not necessarily represent those of their affiliated organizations, or those of the publisher, the editors and the reviewers. Any product that may be evaluated in this article, or claim that may be made by its manufacturer, is not guaranteed or endorsed by the publisher.
